# Editorial: CARMA Proteins: Playing a Hand of Four CARDs

**DOI:** 10.3389/fimmu.2019.01217

**Published:** 2019-05-29

**Authors:** Frédéric Bornancin, Andrew L. Snow

**Affiliations:** ^1^Autoimmunity, Transplantation and Inflammation, Novartis Institutes for BioMedical Research, Basel, Switzerland; ^2^Department of Pharmacology and Molecular Therapeutics, Uniformed Services University of the Health Sciences, Bethesda, MD, United States

**Keywords:** CARD9, CARD10, CARD11, CARD14, Bcl10, Malt1, CARMA, immunodeficiency

Over the past 20 years, enhanced analyses of tumor-specific genomic alterations coupled with elegant biochemical approaches have helped to map essential signaling pathways in healthy and malignant cells. For example, the B cell lymphoma/leukemia 10 (BCL10) gene was identified in 1999 from a recurrent chromosomal translocation noted in non-Hodgkin lymphomas that arise in mucosa-associated lymphoid tissue (MALT). These studies demonstrated that BCL10 could oligomerize via its caspase recruitment domain (CARD) and induce robust activation of nuclear factor of kappaB (NF-κB), a critical family of transcription factors first implicated in lymphocyte differentiation. In <3 years, multiple groups discovered that BCL10 must partner with one of four CARD-containing scaffold proteins (CARD9, CARD10, CARD11, and CARD14) and the MALT1 paracaspase (also discovered from a MALT lymphoma-derived chromosomal translocation) to drive NF-κB signaling in response to various receptor-mediated inputs. Building from murine knockout models, a growing spectrum of malignancies and human immune disorders have been genetically linked to altered signaling through this “CARD-BCL10-MALT1 (CBM) complex.” As this Research Topic attests, indeed this list has expanded exponentially in just the past few years. Drawing from leading investigators in the field, including many involved in the seminal discoveries summarized above, the 10 reviews, three original research articles, and one case report compiled in this Frontiers in Immunology eBook provide a comprehensive and timely examination of CBM complex assembly and signaling. Although we now appreciate that tissue-specific expression of CARMA/CARD proteins dictates function and underlying pathology of associated diseases, this collection also underscores the ubiquity and significance of the CBM signalosome as a central governor of receptor-mediated signaling to NF-κB and additional outputs important for cell proliferation, survival, differentiation, and function.

Two reviews provide an in-depth look at the complex regulation and function of the two subunits shared by all CBM complexes: BCL10 and MALT1. Gehring et al. discuss the discovery and subsequent characterization of BCL10 as the central adaptor protein that channels various innate inflammatory and adaptive antigen receptor stimuli to NF-κB and c-Jun N-terminal kinase (JNK) activation in various immune cells. The formation of CARD-dependent cytoplasmic BCL10 filaments, “seeded” by CARD/CARMA scaffold proteins, constitute an essential platform for MALT1 protease activation and downstream signaling. Informed by studies of T and B cell receptor signaling in lymphocytes, the authors also review the complex post-translational modifications that ensure the orderly extension and degradation of putative BCL10 filaments *in vivo* that ultimately tune and regulate immune responses. Juilland and Thome provide an up-to-date overview of MALT1, which has been intensively interrogated in recent years as a potential therapeutic target for both lymphoma and autoimmune disease. MALT1 constitutively associates with BCL10, and functions both as a scaffold and protease activated upon CBM assembly and BCL10 filament construction. At least 10 MALT1 substrates have been described to date, with more likely to be identified in future studies. Proteolytic cleavage of many of these proteins (e.g. A20, RelB), including MALT1 itself, typically serves to prolong canonical NF-κB activation, although other MALT1 substrates are implicated in regulating cellular adhesion, mRNA stabilization, and metabolic reprogramming in lymphocytes. Both reviews discuss how loss or mutation of BCL10 or MALT1 contribute to immune deficiency and dysregulation, highlighting the need for further mechanistic studies that will better define the essential functions these proteins fulfill in propagating receptor-directed signals in immune and non-immune cells, and maintaining vigorous yet controlled immune responses.

BCL10 and MALT1 are rendered operational primarily through interactions with multi-domain scaffold proteins known as CARD-MAGUK 1-3 (CARMA1-3) and CARD9. These molecules were initially identified based on high sequence similarity shared between their CARD motifs and that of BCL10, facilitating heterotypic CARD-CARD interactions between the two. As illustrated in several salient articles collected in this eBook, these four CARD/CARMA proteins are structurally and functionally homologous in nucleating CBM complexes for downstream signaling. However, their discrete tissue-specific expression patterns dictate the upstream receptors through which these signals are conveyed in different cell types, resulting in distinct functions that help to shape immunity, inflammation, and tumorigenesis.

Two reviews examine the fundamental role of CARD9 in microbial recognition by myeloid lineage cells of the innate immune system. CARD9 is utilized by several membrane and intracellular pattern recognition receptors specific for molecules derived from both commensal and pathogenic bacteria, fungi and viruses. Based on recent studies of human patients with CARD9 deficiency, Drummond et al. discuss the indispensable function of CARD9 in human anti-fungal immunity. CARD9 facilitates CBM-dependent activation of NF-κB, activation of ERK, and production of multiple pro-inflammatory cytokines upon recognition of fungal cell wall components by SYK-coupled C-type lectin receptors such as Dectin-1. Animals and humans lacking CARD9 are particularly susceptible to pathogenic fungal infections of the oral mucosa, skin, and central nervous system, as well as certain bacterial and viral infections. The authors broadly discuss the multi-faceted role of CARD9 in innate immunity, autoimmune disease, and cancer in humans, informed by both population-based studies and rare variants in patients suffering from uncontrolled, spontaneous fungal infections. This review is complimented by original research from De Bruyne et al. describing a *CARD9* founder mutation in a cohort of 11 patients from five Turkish families suffering from various fungal infections. Consistent with a previous report, the authors demonstrate that this specific CARD9 variant (p.Arg70Trp, R70W) fails to stimulate NF-κB. Furthermore, CARD9 R70W also dominantly interferes with WT CARD9 signaling by disrupting BCL10 filament formation and attenuating NF-κB activity, although enough signaling may be preserved to explain the absence of pathology in heterozygous carriers. Remarkably, this dominant negative CARD9 mutant could also repress constitutive NF-κB signaling induced by gain-of-function variants of CARD10, CARD11, and CARD14. Although CARD9 is not co-expressed with these proteins in normal cells, this result beautifully illustrates the evolutionarily conserved requirements for scaffold multimerization and heterotypic CARD-CARD interactions in nucleating signal-competent CBM signalosomes.

In contrast, the review from Hartjes and Ruland focuses primarily on the importance of CARD9 signaling in maintaining homeostasis and a healthy microbiome in the gut. Over the past 10 years, single nucleotide polymorphisms in the human *CARD9* gene have been shown to influence susceptibility to inflammatory bowel disease (IBD) and colorectal cancer development. A detailed overview of several studies using murine colitis models reveals how loss or perturbation of Card9 signaling can alter the composition and function of resident myeloid cell populations and contribute to microbial dysbiosis, intestinal inflammation, and tumorigenesis. Future studies that delineate CARD9-dependent signaling pathways in greater molecular detail will hopefully open new therapeutic avenues for treating invasive fungal infections, IBD and colon cancer.

Four articles in this collection highlight CARD11 (a.k.a. CARMA1) as a critical regulator of adaptive immune responses. The expression of CARD11 is largely restricted to lymphocytes, where it primarily functions to bridge antigen recognition through T and B cell receptors (TCR/BCR) with downstream activation of NF-κB, JNK, and mTOR. Bedsaul et al. review the complex events surrounding TCR/BCR-induced CARD11 signaling. This process involves an elaborate intramolecular regulation of closed and open CARD11 protein conformations, which dictates recruitment and subsequent modification of BCL10, MALT1, and additional co-signaling proteins that serve to either activate the IκB kinase (IKK) complex (e.g., TRAF6, caspase 8) or terminate CARD11 signaling (e.g., GAKIN, PP2A). A brief overview of human somatic and germline *CARD11* mutations found in lymphoma and immunodeficient patients, respectively, culminates in a list of salient unanswered questions about CARD11 signaling that prompt further structure-function studies. A clever original article by Seeholzer et al. answers this call by implementing a creative approach to investigate the molecular details of CBM assembly in lymphocytes. A fusion protein joining BCL10 directly to the N-terminus of CARD11 promotes chronic MALT1 protease activation in CARD11-deficient cell lines, with differential effects on downstream NF-κB activation in B vs. T cells. Structure-guided mutagenesis of either CARD domain within the BCL10-CARD11 fusion protein helped to clarify how BCL10-MALT1 recruitment to CARD11 and formation of BCL10 CARD-dependent filaments cooperate in a highly integrated process for CBM signaling.

Lu et al. build upon this foundation by providing an exhaustive review of recently described human congenital immune disorders attributed to inherited mutations in *CARD11, BCL10*, and *MALT1*. As expected, null mutations in all three genes result in severe combined immunodeficiency, underscoring the importance of CBM signaling for mature B and T cell functions. However, various missense mutations, insertions, and/or deletions that preserve CARD11 protein expression contribute to a surprising spectrum of disease presentations in human patients. Whereas, gain-of-function (GOF) *CARD11* mutations congruent with those found in B cell lymphoma cause a selective B cell lymphoproliferative disorder, loss-of-function (LOF)/dominant negative (DN) *CARD11* variants were recently discovered in patients with severe atopic disease. The authors compare and contrast the expanding list of phenotypic manifestations described in these patients, revealing shared features of immunodeficiency (e.g., poor humoral responses, susceptibility to certain viral infections) that relate back to dysregulated CBM-dependent signaling to NF-κB and mTOR. An intriguing case report offered by Desjardins et al. adds to this complicated picture by challenging the conventional classification of a single *CARD11* mutation as gain or loss-of-function. This study describes a four-generation family that exhibits both B cell lymphocytosis and atopy, linked to a unique inherited *CARD11* variant (p.His234_Lys238delinsLeu) that exhibits both GOF and DN activity in T cell signaling assays. Collectively, these articles describe how *CARD11* mutations can manifest in distinct and blended features of immune dysregulation, depicting the CARD11 scaffold as a critical fulcrum for balanced T and B cell responses.

Two reviews delve into CARD14/CARMA2 as the pivotal scaffold for CBM signaling in epithelial cells and keratinocytes, focusing on how dysregulation of CARD14 signaling contributes to inflammatory skin disorders such as psoriasis. CARD14/CARMA2 was perhaps the least characterized member of the CARMA/CARD protein family, but recent insights have illuminated how dysregulation of CARD14 can result in skin inflammation. Zotti et al. consider how three alternative splice variants of CARD14 affect NF-κB triggering in response to pathogen-associated molecular patterns (and stimuli yet to be identified), with a short CARMA2 isoform (CARMA2*sh*) lacking the C-terminal SH3 and guanylate kinase (GUK) domains predominating in human skin. Various GOF CARMA2*sh* variants linked to psoriasis, pityriasis and other skin disorders are also discussed in relation to an amplified pro-inflammatory NF-κB-dependent gene program in the skin. Importantly, the authors also discuss emerging roles for several proteins in enabling (e.g., TRAF2, DEPDC7) or modulating (e.g., ULK2, RNF7) CARD14 signaling, which may present novel avenues for therapeutic intervention. The article by Israel and Mellett presents a deeper dive into the molecular genetics and clinical features of inflammatory skin disorders caused by numerous human *CARD14* mutations described to date. Informed by detailed phenotyping and clinical management of patient kindreds, mouse models of psoriasis harboring synonymous *Card14* mutations, and *in vitro* molecular signaling studies, this review summarizes how different human *CARD14* variants can manifest in a heterogeneous spectrum of psoriatic skin diseases. Biologic therapies targeting TNF and the IL-12/IL-23/IL-17 cytokine axis have already proven beneficial in treating these diseases, whereas future specific inhibitors of CARD14 or MALT1 protease may prove to be attractive tools for tamping down inflammation driven by CARD14 GOF mutations.

In contrast to the other CARD/CARMA proteins, CARD10/CARMA3 is ubiquitously expressed in many non-lymphoid tissues and may play an important role in the pathogenesis of several types of cancer. Two salient reviews explore this emerging paradigm, offered from investigators that have led the characterization of CARD10 signaling since its initial discovery. Zhang and Lin provide a general overview of CARD10/CARMA3 in CBM signaling induced by multiple G-protein coupled receptors and receptor tyrosine kinases (RTKs) such as the epidermal growth factor receptor (EGFR), culminating in NF-κB activation in several cell types. The authors also discuss newly recognized partners in CARD10 signaling (e.g., TMEM43), as well as a recently uncovered role for CARD10 in the antiviral interferon response. McAuley et al. discuss knowns and unknowns about how CARD10-dependent signals downstream of a variety of GPCR/RTKs promote solid tumor pathogenesis in non-immune cells. CARD10 overexpression has been noted in many tumor types, affected in part by failed miRNA-mediated modulation. Both reviews also comment on recent reports of receptor-independent CBM signaling in response to DNA damage, and reminds readers of the many unanswered questions surrounding the mechanisms connecting CARD10-dependent CBM signaling to various receptors, and how these might be specifically targeted for anti-cancer therapy.

Finally, an original article from Staal et al. describes the ancient evolutionary origin of CBM signaling in invertebrates, revealing co-evolution of CARD-CC homologs with Bcl10, Syk-like kinases, and type I paracaspases. Careful structure/function analysis of paracaspases from invertebrates and lower vertebrates suggests that MALT1 functions for NF-κB activation evolved recently in jawed vertebrates, despite the appearance of conserved, independent scaffold and protease functions in older invertebrates. Their results highlight the utility of a comparative biology approach for studying CBM complex functions, and point to fascinating alternative CBM-independent roles for MALT1, including neuronal function in *C. elegans*.

In summary, the articles and reviews in this eBook constitute the most comprehensive, current assessment of CBM complex function in normal and pathologic settings. We hope this collection will inspire future mechanistic studies to identify novel therapeutic approaches to treating myriad diseases connected to dysregulation of this ubiquitous and critical signalosome.

## Author Contributions

All authors listed have made a substantial, direct and intellectual contribution to the work, and approved it for publication.

### Conflict of Interest Statement

FB is an employee and shareholder of Novartis. The remaining author declares that the research was conducted in the absence of any commercial or financial relationships that could be construed as a potential conflict of interest.

